# Effect of new-style anterior and posterior vaginal wall repair combined with modified ischial spine fascia fixation on patients with pelvic organ prolapse and their postoperative quality of life

**DOI:** 10.3389/fsurg.2022.994615

**Published:** 2022-10-06

**Authors:** Gensheng Wang, Shengju Zhou, Shuhua Wang, Dongdi Xu, Dan Wang, Hongling Xu, Chuanlong Gao, Qing Li

**Affiliations:** ^1^ Department of Obstetrics and Gynecology, Anqing Hospital Affiliated to Anhui Medical University, Anqing, China; ^2^ Department of Obstetrics and Gynecology, Anqing Hospital Huining Branch Affiliated to Anhui Medical University, Anqing, China; ^3^ Department of Obstetrics and Gynecology, Qianshan Municipal Hospital, Qianshan, China

**Keywords:** anterior and posterior vaginal wall repair, ischial spine fascia fixation, effect, pelvic organ prolapse, pop

## Abstract

**Objective:**

This study aims to explore the effect of new-style anterior and posterior vaginal wall repair combined with modified ischial spine fascia fixation on patients with pelvic organ prolapse (POP) and their postoperative quality of life.

**Methods:**

A total of 88 patients with POP and elective surgery admitted to Anqing Hospital affiliated to Anhui Medical University from March 2018 to March 2021 were retrospectively analyzed. According to their surgical methods, patients were divided into an observation group [44 cases, all underwent new-style anterior and posterior vaginal wall repair combined with modified ischial spine fascia fixation (new-style APVR-modified ISFF)] and a control group [44 cases, all underwent traditional anterior and posterior vaginal wall repair combined with sacrospinous ligament fixation (traditional APVR- SLF)]. The perioperative indicators were compared between the two groups. The pelvic floor function, pelvic organ prolapse quantification (POP-Q) classification, and quality of life were observed before operation, 3 months after operation, and 6 months after operation. All patients were followed-up.

**Results:**

Compared with the control group, the observation group had more advantages in intraoperative blood loss, operation time, urinary catheter indwelling time, postoperative anal exhaust time, and hospitalization time (*P* < 0.05). In terms of pelvic floor function, patients of both groups showed significant improvement at 3 months and 6 months after surgery (*P* < 0.05). In terms of quality of life, the two groups exhibited significant improvement at 6 months after surgery (*P* < 0.05). PFIQ-7, PFDI-20, and UDI-6P of the observational group were lower than those of the control group, while PISQ-12 was higher than that of the control group but all with no significant difference (*P* > 0.005). In addition, the total complication rate of the observation group was 2.27% (1/44), which was significantly lower than 22.73% (10/44) of the control group (*P *< 0.05).

**Conclusion:**

New-style APVR-modified ISFF can effectively treat POP and improve the quality of life of such patients, with less postoperative complications and high safety.

## Introduction

Female pelvic organ prolapse (POP) is a common chronic disease in clinical practice, which occurs in 15%–30% population around the world and 40% in China (1–2). It can occur in a single site or in a combination of two or more parts, of which the vaginal wall and uterine are the organs that take the majority (3). At present, only nonsurgical treatments such as pessary and pelvic floor rehabilitation training are performed on patients with mild POP, while surgical treatment is performed on patients with severe POP (4).

Conventional vaginal hysterectomy plus vaginal wall repair in surgical treatment cannot completely solve the problem of pelvic prolapse. Ischial spine fasciae fixation and sacrospinous ligament fixation are the common surgical methods in clinical practice for pelvic prolapse due to their good tensile force and no need for mesh (5). In sacrospinous ligament fixation, the sacrospinous ligament is difficult to expose due to its position and shallow suture, which easily causes recurrence of postoperative prolapse, while sacral fascia fixation has complications of intestinal wall damage and bleeding (6). Therefore, how to reduce complications and recurrence rate after surgery while improving the treatment effect of patients with POP is a problem that needs to be solved urgently. The team at the Pelvic Floor Dysfunction Diagnosis and Treatment Center of Peking University Shenzhen Hospital innovated this surgical method, and by changing the surgical approach and knotting method of sacral spine fascia fixation, the improved ischial spine fascia fixation was applied to the clinic, and great results had been achieved (7).

Therefore, this study aimed to explore the effect of new-style anterior and posterior vaginal wall repair combined with modified ischial spine fascia fixation (new-style APVR-modified ISFF) on patients with vaginal wall and uterine after surgery and their quality of life (QoL).

## Materials and methods

### Subjects

A total of 88 patients with POP and elective surgery admitted to Anqing Hospital affiliated to Anhui Medical University from March 2018 to March 2021 were retrospectively analyzed. The patients were divided into an observation group and a control group according to the different surgical methods.

This study was approved by the Ethics Committee of Anqing Municipal Hospital.

Inclusion criteria are as follows: (1) patients who met the diagnostic criteria of POP (8) with Grade III–IV POP quantification (POP-Q) (9); (2) patients without fertility requirements; (3) patients without contraindications related to surgery and anesthesia; and (4) patients who signed an informed consent form.

Exclusion criteria are as follows: (1) patients who received pelvic floor physical therapy within 3 months before surgery; (2) patients with severe primary diseases of the heart, brain, liver, kidney, and other important organs; (3) patients with malignant tumors of the rectum, ovary, uterus, and other pelvic cavity requiring surgical treatment; (4) patients who have undergone relevant surgical treatment in the past; (5) patients with poor treatment compliance; and (6) patients who cannot be followed-up regularly.

### Surgical methods

Anesthesia method: All patients were treated with epidural anesthesia after completing a preoperative examination and taking a lithotomy position.

Transvaginal hysterectomy: Two branches of hypophysin + 200 ml of normal saline were injected into the fornix of the cervix to stop the bleeding. A transverse incision was made 5 mm below the sulcus of the bladder and cervix, the vaginal fornix to the cervical fascia layer was incised, and the vesicocervical space and the rectocervical space were gradually separated. The re-entrant peritoneum was opened, the uterosacral-cardinal ligament on both sides were sutured, and coagulotomy were performed in the uterine blood vessels, broad ligaments and proper ovarian ligaments on both sides. All patients would receive a hysterectomy.

**Figure 1:**
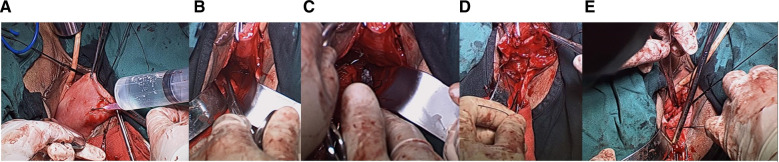
Intraoperative illustration of patients in the control group. (**A**) The vaginal wall is incised, and the gap is separated to the sciatic spinous ligament; (**B**) the space around the sacrospinous ligament is separated to expose the sacrospinous ligament; (**C**) the sacrospinous ligament is sutured about 2 cm inside the sacrospinous ligament; (**D**) the other end is sutured on the fascia of the posterior vaginal wall and does not penetrate the posterior vaginal wall; (**E**) the vaginal fornix is raised after knotting the sutures.

The patients in the control group all underwent traditional anterior and posterior vaginal wall repair combined with sacrospinous ligament fixation (traditional APVR-SLF). The right main sacral ligament suture was buried under the mucosa of the posterior vaginal wall, the anterior and posterior vaginal wall stumps were sutured, the posterior vaginal wall was clamped at the junction of skin and mucous membranes (1 cm below it and 2 cm in the vaginal opening), and water was injected into the submucosal wall for separation. Between the two forceps, the mucosa of the posterior vaginal wall was longitudinally incised for 3 cm, and the right rectal space was separated to reach the level of the ischial spine. The ischial spine fascial fixation as performed by using a No. 7 thread to suture the fascia 1 cm above the ischial spine. After all the tension sutures had been used, the main sacral ligament buried in the submucosa of the posterior vaginal wall was removed and tied with the sciatic fascia or sacrospinous ligament sutures, and the posterior vaginal wall was sutured (Figure 1).

**Figure 2:**
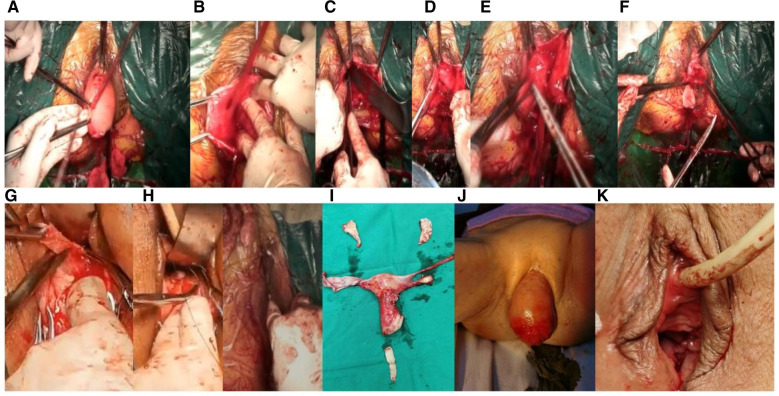
Intraoperative pictures of patients in the observation group. (**A**) After hysterectomy, the vaginal mucosa in front of the bladder is incised; (**B**) the vaginal bladder space is separated by an anterior approach to the ischial spine and the fascia 1 cm above it; (**C**) nonabsorbable suture are used to suture the ischial spine fascia; (**D**) pull the suture and try to pull the force; (**E**) the other end of the suture is fixed on the inner vaginal fascia, do not penetrate the vaginal mucosa, and the suture is not knotted temporarily; (**F**) trim part of the anterior vaginal wall mucosa and purse-string suture the bladder; (**G**) trim part of the posterior vaginal wall to the top of the vaginal fornix; (**H**) suture the posterior wall of the vagina, narrow and fasten the vagina; (**I**) after the anterior and posterior walls of the vagina are sutured, the sciatic fascia sutures and the sutures at both ends of the vaginal fascia are knotted to fix the sutures at both ends of the vaginal fascia to lift the vaginal vault; (**J**) whole uterus, part of vaginal wall specimen; (**K**) preoperative photo of pelvic organ prolapse; (**L**) postoperative photo.

Patients in the observation group underwent new-style APVR-modified ISFF. The anterior vaginal wall was cut longitudinally along the midline, up to the inferior urethral groove, down to the anterior fornix of the vagina, and on both sides to the level of the descending pubic branch, fully separating the bladder from the vaginal wall. After removing the prolapsed uterus first, entry was made from the vaginal bladder space. The position of the right sciatic spine was touched with fingers, and the first stitch at the inner side of the sciatic spine (1–1.5 cm) was sutured. One end of No. 7 silk thread was sutured to the sacrospinous ligament of the vaginal vault or cervix, and the other end was sutured to the inner side of the right ischial fascia (1.0 cm). The suture depth was 0.3–0.5 cm (Figure 2).

The resection range of the posterior wall of the vagina was an equilateral triangle from the vaginal opening to the lower one-third of the vagina, and the apex of the equilateral triangle was up to the posterior fornix of the vagina as a distance. After suturing, a longitudinal scar was formed on the midline of the vagina. The incision on the front and rear walls of the vagina was sutured to tighten the entire vagina. In the process of closing the front and rear walls of the vagina, the ends of the reserved sutures were threaded through the fascia in the vaginal wall at the right corner of the top of the vagina and the knot was tied to lift the vaginal roof.

Rehabilitation after operation: urinary catheter was indwelled for 24 h after the operation, gauze was applied to the vagina to stop bleeding, and antibiotics were given for 48–72 h. Patients were informed to avoid chronic coughing, weight lifting, constipation, and other conditions that can lead to increased chronic abdominal pressure after the operation. The patients were advised to avoid weight-bearing for the next year, keep the perineum clean and dry, pay attention to rest, strengthen nutrition, refrain from sexual intercourse, and bathe for 3 months.

### Observation index

Perioperative-related indicators: These include intraoperative blood loss, operation time, urinary catheter indwelling time, postoperative anal exhaust time, and hospitalization time.

Pelvic floor function: The pelvic floor function of two groups was compared before operation, 3 months after operation, and 6 months after operation. According to the POP quantification (POP-Q) proposed by Professor Bump (9), the anatomical recovery of each point in the pelvic cavity should be evaluated when holding the breath downward. The POP-Q system stipulates that the degree of POP should be determined according to the measurement results of three anatomical markers , including genital hiatus (GH) length, perineal body (PB) length, and total vaginal length (TVL), and six points. The midline of the anterior vaginal wall is 3 cm from the edge of the hymen (Aa), the anterior vaginal wall prolapse is the farthest from the hymen (Ba), and the midline of the posterior vaginal wall is 3 cm from the edge of the hymen (Ap) and the posterior vaginal wall. The prolapse is the farthest from the hymen (Bp), the hymen reference point (C), and the distance from the posterior vaginal fornix to the hymen (D). The POP-Q scores of the two groups were compared 1 month after operation.

Quality of life: The quality of life before and 6 months after operation was compared between the two groups. The quality of life includes the impact on pelvic floor function, pelvic floor dysfunction, urination, and quality of sexual life. The Pelvic Floor Impact Questionnaire-Short Form 7 (PFIQ-7) score was used to evaluate the effect of pelvic floor function (10); the higher the score, the worse the pelvic floor function. Pelvic floor dysfunction was evaluated by the Pelvic Floor Distress Inventory-Short Form 20 (PFDI-20) score (11); the higher the score, the more obvious the pelvic floor disorder. Urination was evaluated by the Urinary Distress Inventory (UDI-6) score (12); the higher the score, the worse the urination function. The quality of sexual life was assessed using the POP-Urinary Incontinence Sexual Questionnaire 12 (PISQ-12) score (13). The full score of the scale was 48 points, including sexual partners, emotional factors, physiological factors, and other items. The higher the score, the better the quality of sexual life.

Follow-up: The postoperative complications such as hematoma in the rectal space, incision infection, and urinary retention were recorded in the two groups. Statistics of recurrence within 1 year was analyzed.

### Statistical methods

All the data collected in this study were analyzed using SPSS 21.0 software. Normally distributed measurement data were expressed as mean ± standard deviation (SD), while non-normally distributed measurement data were expressed as median (interquartile range), and the comparisons were examined by Student’s *t*-test. The categorical data were expressed as *n*(%), and the differences between the two groups were examined by chi-square analysis or Fisher's exact test. *P* < 0.05 was considered statistically significant.

## Results

There were 44 patients in the observation group with an average age of 59.79 ± 7.87 years (45–75) and 44 patients in the control group with an average age of 59.81 ± 7.58 year (45–76). There was no difference in age, pregnancy time, prolapse location, prolapse scale, and comorbidities between the two groups (*P* > 0.05) (Table 1).

**Table 1:** Comparison of general information between two groups of patients.

Index	Observation group (*n* = 44)	Control group (*n* = 44)	*P*-value
Average age (years)	59.8 ± 7.9	59.8 ± 7.6	0.990
BMI (kg/m^2^)	26.03 ± 2.39	25.98 ± 2.28	0.921
Pregnant number (times)	3.76 ± 0.21	3.71 ± 0.19	0.245
Birthing number (times)	2.45 ± 0.23	2.42 ± 0.21	0.525
Location			
Uterine prolapse (*n*)	43 (97.73%)	42 (95.45%)	0.557
Posterior vaginal wall prolapse (*n*)	39 (88.63%)	36 (81.82%)	0.367
Anterior vaginal wall prolapse (*n*)	44 (100.00%)	43 (97.73%)	0.315
POP-Q			
III (*n*)	39	40 (90.91%)	0.526
IV (*n*)	5	4 (9.09%)	
Average front wall point A value (cm)	1.73 ± 0.23	1.71 ± 0.22	0.678
Average front wall point B value (cm)	3.87 ± 0.43	3.82 ± 0.41	0.578
Combined disease			
Hypertension (*n*)	18 (40.91%)	20 (45.45%)	0.667
Diabetes (*n*)	6 (13.64%)	7 (15.91%)	0.764
Anemia (*n*)	3 (6.82%)	4 (9.09%)	0.694
Past medical history			
Cerebral infarction (*n*)	4 (9.09%)	3 (6.82%)	0.694
Stress incontinence (*n*)	3 (6.82%)	2 (4.55%)	0.645

BMI, body mass index; POP-Q, pelvic organ prolapse quantification.

Compared with the control group, the observation group had less intraoperative blood loss (65.82 ± 3.98 vs. 88.98 ± 4.21 ml), less operation time (82.98 ± 3.88 vs. 89.08 ± 3.81 min), less urinary catheter indwelling time (2.13 ± 0.12 vs. 2.87 ± 0.13 days), less postoperative anal exhaust time (24.32 ± 0.24 vs. 26.87 ± 0.28 h), and short hospital stay (4.21 ± 0.32 vs. 5.87 ± 0.37 days) (all *P*’s < 0.001) (Table 2).

**Table 2:** Comparison of indexes in the perioperative period between the two groups.

Index	Observation group (*n* = 44)	Control group (*n* = 44)	*P*
Intraoperative blood loss (ml)	65.82 ± 3.98	88.98 ± 4.21	<0.001
Operation time (min)	82.98 ± 3.88	89.08 ± 3.81	<0.001
Catheter indwelling time (d)	2.13 ± 0.12	2.87 ± 0.13	<0.001
Postoperative anal exhaust time (h)	24.32 ± 0.24	26.87 ± 0.28	<0.001
Hospital stay (d)	4.21 ± 0.32	5.87 ± 0.37	<0.001

Before the operation, there was no difference in Aa, Ba, C, Ap, Bp, and TVL between these two groups (all *P*’s > 0.05). However, Aa, Ba, C, Ap, Bp, and TVL of these two groups were significantly improved after 3 and 6 months when compared with their values before the operation (all *P* < 0.05). No difference was found between these two groups of all indexes mentioned above, both 3 and 6 months after surgery (*P* > 0.05) (Table 3).

**Table 3:** Comparison of pelvic floor function between the two groups.

Index	Observation group (*n* = 44)	Control group (*n* = 44)	*P*-value
Aa (cm)			
Preoperative	2.42 ± 0.67	2.40 ± 0.56	0.880
3 months postoperation	−1.23 ± 0.21*	−1.19 ± 0.23*	0.397
6 months postoperation	−2.54 ± 0.21*	−2.49 ± 0.24*	0.301
Ba (cm)			
Preoperative	2.11 ± 0.45	2.09 ± 0.48	0.840
3 months postoperation	−1.56 ± 0.39*	−1.62 ± 0.32*	0.432
6 months postoperation	−2.75 ± 0.34*	−2.69 ± 0.36*	0.424
C (cm)			
Preoperative	2.39 ± 0.32	2.35 ± 0.36	0.583
3 months postoperation	−4.78 ± 0.34*	−4.71 ± 0.32*	0.323
6 months postoperation	−6.11 ± 0.37*	−6.01 ± 0.33*	0.184
Ap (cm)			
Preoperative	2.59 ± 0.32	2.54 ± 0.37	0.500
3 months postoperation	−1.83 ± 0.29*	−1.76 ± 0.27*	0.244
6 months postoperation	−1.74 ± 0.26*	−1.71 ± 0.23*	0.568
Bp (cm)			
Preoperative	2.49 ± 0.37	2.42 ± 0.33	0.351
3 months postoperation	−1.65 ± 0.28*	−1.61 ± 0.25*	0.481
6 months postoperation	−2.45 ± 0.25*	−2.39 ± 0.22*	0.235
TVL (cm)			
Preoperative	8.21 ± 0.46	8.17 ± 0.41	0.668
3 months postoperation	7.87 ± 0.43*	7.93 ± 0.47*	0.534
6 months postoperation	7.82 ± 0.41*	7.87 ± 0.45*	0.587

Compared with before operation, *P < 0.05.

Aa, which is 3 cm from the midline of the anterior vaginal wall to the edge of the hymen, corresponding to the “bladder urethra fold.”

Ba is the reflex of the anterior fornix of the vagina or the farthest position of the vaginal stump from point Aa.

C is the intact uterus representing the farthest part of the external cervix, and the hysterectomy is equivalent to the vaginal stump.

Ap is 3 cm from the midline of the posterior vaginal wall to the edge of the hymen.

Bp is the reflex of the posterior fornix of the vagina or the farthest distance from the Ap point of the vagina stump.

TVL, Total vaginal length: the total length from the top of the vagina to the edge of the hymen when C and D are in the normal position.

No difference was found in the POP-Q scale between the two groups (*P* > 0.05) (Table 4).

**Table 4:** Comparison of the POP-Q score between two groups.

POP-Q	Observation group (*n* = 44)	Control group (*n* = 44)	*P*-value
0°	41 (93.18%)	38 (86.36%)	0.291
I°	2 (4.55%)	4 (9.09%)	0.398
II°	1 (2.27%)	2 (4.55%)	0.557

POP-Q, pelvic organ prolapse quantification.

In addition, there was no difference in the PFIQ-7 score, PFDI-20 score, UDI-6P score, and PISQ-12 score between the two groups before surgery (*P* > 0.05). However, these indexes significantly improved in both groups 6 months after surgery (all *P*’s < 0.05). No difference was found between the two groups in all indexes mentioned above 6 months after surgery (all *P*’s > 0.05) (Table 5).

**Table 5:** Comparison of the quality of life between the two groups.

Index	Observation group (*n* = 44)	Control group (*n* = 44)	*P*-value
PFIQ-7			
Preoperative	86.92 ± 2.98	86.89 ± 3.01	0.963
6 months postoperation	13.28 ± 2.87*	14.26 ± 2.85*	0.112
PFDI-20			
Preoperative	16.98 ± 1.09	16.72 ± 1.12	0.273
6 months postoperation	7.52 ± 1.21*	8.01 ± 1.19*	0.059
UDI-6P			
Preoperative	15.76 ± 1.15	15.72 ± 1.12	0.869
6 months postoperation	5.38 ± 1.02*	5.76 ± 1.05*	0.089
PISQ-12			
Preoperative	19.87 ± 2.01	19.79 ± 2.12	0.856
6 months postoperation	38.98 ± 3.21*	37.78 ± 3.13*	0.079

Compared with before operation, *P < 0.05.

PFIQ-7, pelvic floor impact questionnaire-short form 7; PFDI-20, pelvic floor distress inventory-short form 20; UDI-6, urinary distress inventory; PISQ-12, POP-urinary incontinence sexual questionnaire 12.

There was one (2.27%) case with hip pain 1–2 days after surgery in the observational group. No vaginal and vulvar drop feeling and vaginal bulging feeling were observed in the observational group. The complication rate of the control group was 22.73% (10 cases), including 1 (2.27%) case with rectal hematoma, 1 (2.27%) case with incision infection, 3 (6.82%) cases with urinary retention, and 5 (11.36%) cases with hip pain. The total incidence of postoperative complications in the observation group was significantly lower than that in the control group (1 vs. 10 cases, 2.27% vs. 22.73%) (*P* = 0.004) (Table 6).

**Table 6:** Complications of patients after surgery.

Index	Observation group (*n* = 44)	Control group (*n* = 44)	*P*-value
Hip pain	1 (2.27%)	5 (11.36%)	
Rectal hematoma	0 (0.00%)	1 (2.27%)	
Incision infection	0 (0.00%)	1 (2.27%)	
Urinary retention	0 (0.00%)	3 (6.82%)	
Total	1 (2.27%)	10 (22.73%)	0.004

The median follow-up time of all patients in this study was 20 months (12–24 months). During the operation in both groups, no damage to adjacent organs, such as the urethra, bladder, and ureter, and no recurrence were found during the follow-up.

## Discussion

POP is defined as the abnormal descent of pelvic organs from their normal position or disorder of the pelvic tissue, which induces its dysfunction. POP often occurs under conditions of increased pelvic and abdominal pressure or gravity, which has a severe impact on women's daily life (14). Vaginal delivery, prolific birth, vaginal genital area atrophy, pelvic connective tissue disorder, heavy physical labor, neuropathy, and so on are the risk factors for this disease (15). At present, it is generally believed that the pathogenesis of the disease is the “Hammock Hypothesis,” “Three Levels” theory, “Three Chamber System,” and “Integral Theory” (16). The pelvic floor tissue is artificially divided into three different levels, or the pelvic cavity is divided into three areas. POP-related diseases often involve dysfunctioned or prolapsed organs with more than one of these tissues (16). This is the fundamental reason there are so many types of POP surgeries, and the pros and cons coexist (17–19). Previous studies have shown that the “repair and reconstruction” of organs is to achieve the best curative effect through minimally invasive surgery and to explore more economical, practical, and easy-to-promote treatment methods (20).

Transvaginal hysterectomy is currently the most commonly used procedure for treating POP, which can reconstruct and restore the original function of the pelvic cavity. Still, it is difficult to achieve satisfactory results alone (21). Previous studies have shown that transvaginal hysterectomy combined with anterior and posterior vaginal wall repair can reduce the damage to the surrounding tissues of patients with POP, which effectively alleviates the clinical symptoms of patients (22). Both the ischial spine fascia fixation and sacrospinous ligament fixation use the ligaments and fascia tissues of patients as supporting structures for pelvic reconstruction operations, which can avoid erosion and exposure induced by the use of mesh and other problems, which can also achieve satisfactory results (23). The two groups of surgery in this study adopted the natural orifice operation through the vagina, which could repair the pelvic floor at three levels at the same time and the anterior and posterior vaginal walls. The surgery could be done under the condition of intraspinal anesthesia to reduce the risk of cardiovascular and cerebrovascular complications caused by general anesthesia and is especially suitable for elderly patients with comorbidity (24–25). The ischial spine is an important anatomical landmark for two operations due to the attachment of the sacrospinous ligament, levator tendon arch, pelvic fascia tendon arch, and obturator aponeurosis, which locates 1 cm outside of the muscle fascia tissue (26). The sacrospinous ligament is a fan-shaped dense connective tissue; its posterior medial side is attached to the lateral edge and front of the sacrum from about the fourth sacral plane to the coccyx, and it is attached to the ischial spine anteriorly and outwardly (26). The suture points of the two kinds of operations are the ischial fascia 1 cm anterior and lateral to the most prominent point of the ischial spine. The sacrospinous ligament is 2.5 cm away from the ischial spine, and they have the same surgical path. The results of the present study showed that the observation group had significantly less intraoperative blood loss, operation time, catheter indwelling time, postoperative anal exhaust time, and length of hospital stay when compared with the control group. These results were also consistent with the research results of Zhiqin (23). This may be due to the easy suture of the superficial position of the ischial spine fascia so that it could shorten the operation time and promote the recovery of the patient.

The POP-Q system was proposed by American scholar Bump and revised by the International Continence Society (ICS), American Urogynecology Society (AUGS), and American Society of Gynecological Surgeons (SGS) (9). A higher stage of POP-Q was considered a risk factor for failure of uterine prolapse surgery (27). The POP-Q scoring system needs to be based on three anatomical markers, length of the genital tract cleft (GH), length of the perineal body (PB), and the total length of the vagina (TVL), and six points, Aa, Ba, Ap, Bp, C, and D (28). Fangli et al. (7) used transvaginal hysterectomy and modified ischial spine fascia to treat 45 cases of POP-Q staging, as II–IV degree POP. The results showed that the new-style APVR-modified ISFF has the advantages of simplicity, safety, improvement of pelvic floor function, relief of prolapse symptoms, high cure rate, and good short-term effect. Yuling et al. (29) performed transvaginal ischial spine fascia fixation suture and sacrospinous ligament fixation on 34 cases of uterine prolapse, and the results showed that modified ischial spine fascia fixation could improve the pelvic floor function of these patients. It had the advantages of a high cure rate, low recurrence rate, and little impact on sexual life after surgery. The results of this study showed that the levels of Aa, Ba, C, Ap, Bp, and TVL were significantly improved after surgery compared with those before. It was suggested that both surgical methods could improve the pelvic floor function of patients with POP.

Previous studies have shown that female sexual function can be restored after improving the symptoms of POP (30). The narrowing of the vaginal vault, the lack of regular uterine contractions, and the lack of orgasm have a serious impact on the quality of the patient's sexual life after the hysterectomy (30). Both the new-style APVR-modified ISFF and traditional APVR-SLF can follow the modern pelvic floor reconstruction principles using their ligaments and fascia tissues as supporting structures for pelvic floor reconstruction surgery, with satisfactory results while avoiding the erosion, exposure, and protrusion caused by the mesh (17). The evaluation of the efficacy of POP surgery should not only refer to anatomical reduction (31) but also sexual function (32). In this study, we paid more attention to the subjective feelings, quality of life, and quality of sexual life of patients. The PFIQ-7 score, PFDI-20 score, UDI-6P score, and PISQ-12 are all questionnaire surveys to evaluate the quality of life of patients with pelvic floor organ prolapse, which are now considered to be important indicators for evaluating the therapeutic effect of POP (33). The results of this study showed that the four quality-of-life scores of these two groups significantly improved at 6 months after surgery compared with those before, suggesting that both surgical methods can improve the quality of life of patients with POP. Of these ways, the QoL improvement in the observational group might be due to the complete separation of the bladder and vagina, attachment of the vaginal dome or uteri to anterior sacrosciatic ligaments, and suture of tissue 1 cm inside the ischial spine fascia, which would increase the support and suspension of the posterior wall of the middle urethra. In addition, a combination of modified ischial spine fascia fixation would be supportive and pertinent for restoration of the bladder, which would improve the QoL of patients. The study of Ren et al. (34) confirmed that ischial spine fascia fixation could effectively reduce the recurrence rate of patients with POP. The results of the present study showed that the total complication rate of the observation group was significantly lower than that of the control group but with no difference in the recurrence rate, suggesting that the new-style APVR-modified ISFF could reduce the complication rate of patients with POP more safely. This might be because the ischial spine fascia is the fascial tissue from the most prominent point of the ischial spine to 1 cm outside. At the confluence of the sacrospinous ligament, the levator tendon arch, the pelvic fascia tendon arch, and the obturator fascia, the tissue is dense, without important blood vessels and nerves, and it is safe and feasible to use this place as the top of the vagina. Fixing the vaginal vault to the ischial spine fascia can restore the anatomical height of the top of the vagina and provide it with effective support (35). In this study, there was one case of hip pain after the new-style APVR-modified ISFF, which occurred 1–2 days after the operation. It might be because the sciatic fascia was relatively far away from the sciatic nerve and the obturator nerve, and the chance of direct damage was relatively small. The buttock pain might be related to the damage to the small nerves innervating the pelvic floor muscles. After giving analgesic treatment, this patient recovered and was discharged.

In addition, there were still several limitations in the present study. First, there was inherent bias in this study due to its retrospective nature. Second, it was a single-center analysis with a small sample; whether the normal pelvic anatomical position could be maintained in the later period should be verified by a large-scale and long-term randomized controlled trial. Thus, all results should be interpreted cautiously.

## Conclusion

In summary, the new-style APVR-modified ISFF can effectively reduce the amount of intraoperative blood loss, accelerate recovery and quality of life, and bring down postoperative complications and recurrence rate.

## Data Availability

The original contributions presented in the study are included in the article/Supplementary Material; further inquiries can be directed to the corresponding author/s.
